# Efetividade e Segurança da Edoxabana nos Cuidados Clínicos de Rotina de Pacientes com Fibrilação Atrial no Brasil: Estudo Prospectivo de Acompanhamento de 1 Ano – EdoBRA

**DOI:** 10.36660/abc.20240589

**Published:** 2025-04-02

**Authors:** Dalton Bertolim Precoma, Rafael Paletta da Silva, Luiz Carlos Santana Passos, Conrado Roberto Hoffman, Fábio Serra Silveira, José Tarcísio Medeiros de Vasconcelos, Sérgio Luiz Zimmermann, Artur Haddad Herdy, Alessandra Ritter, Daniely Freitas-Alves, José Francisco Kerr Saraiva

**Affiliations:** 1 Sociedade Hospitalar Angelina Caron Campina Grande do Sul PR Brasil Sociedade Hospitalar Angelina Caron, Campina Grande do Sul, PR – Brasil; 2 Daiichi Sankyo Brasil Farmaceutica LTDA São Paulo SP Brasil Daiichi Sankyo Brasil Farmaceutica LTDA, São Paulo, SP – Brasil; 3 Universidade Federal da Bahia Salvador BA Brasil Universidade Federal da Bahia, Salvador, BA – Brasil; 4 Hospital Regional Hans Dieter Schmidt Joinville SC Brasil Hospital Regional Hans Dieter Schmidt, Joinville, SC – Brasil; 5 JMF Clínica do Coração Aracaju SE Brasil JMF Clínica do Coração, Aracaju, SE – Brasil; 6 Real e Benemérita Associação Portuguesa de Beneficência São Paulo SP Brasil Real e Benemérita Associação Portuguesa de Beneficência, São Paulo, SP – Brasil; 7 Hospital das Clínicas Faculdade de Medicina Universidade de São Paulo São Paulo SP Brasil Instituto do Coração do Hospital das Clínicas da Faculdade de Medicina da Universidade de São Paulo, São Paulo, SP – Brasil; 8 Universidade Regional de Blumenau Clínica Procardio Blumenau SC Brasil Universidade Regional de Blumenau – Clínica Procardio, Blumenau, SC – Brasil; 9 Instituto de Cardiologia de Santa Catarina São José SC Brasil Instituto de Cardiologia de Santa Catarina, São José, SC – Brasil; 10 IQVIA Brasil São Paulo SP Brasil IQVIA Brasil, São Paulo, SP – Brasil; 11 Pontifícia Universidade Católica de Campinas Campinas SP Brasil Pontifícia Universidade Católica de Campinas, Campinas, SP – Brasil

**Keywords:** Anticoagulantes, Fibrilação Atrial, Hemorragia, Acidente Vascular Cerebral

## Abstract

**Fundamento:**

A edoxabana é um anticoagulante oral que se mostrou segura e eficaz na prevenção de acidente vascular cerebral em pacientes com fibrilação atrial (FA). Dada a ampla utilização da edoxabana desde sua aprovação, é crucial avaliar seu desempenho no contexto clínico brasileiro.

**Objetivos:**

O objetivo do estudo foi descrever a segurança e a efetividade da edoxabana no tratamento de pacientes com FA no Brasil.

**Métodos:**

O EdoBRA é um estudo multicêntrico, prospectivo, observacional, conduzido em 30 centros de pesquisa no Brasil. Eventos de sangramento foram considerados medidas de segurança, e eventos cardiovasculares foram considerados para medidas de efetividade. Análises descritivas foram realizadas. Curvas de Kaplan-Meier foram geradas para análise do tempo para o evento, e um intervalo de confiança de 95% usado conforme apropriado.

**Resultados:**

Entre os 705 pacientes recrutados, 590 foram incluídos na análise por apresentarem pelo menos um evento relatado ou durante o seguimento. As médias (±DP) dos escores de risco CHA2DS2-VASc e HAS-BLED foram, respectivamente, 3 (3,3 ± 1,6) e 2 (1,8 ± 1,2). Durante o acompanhamento de um ano, foram relatados nove sangramentos maiores, incluindo cinco casos de sangramento gastrointestinal [PI 0,85 (IC95% =0,82; 0,88]). Entre os eventos cardiovasculares registrados (n=68), houve quatro eventos de acidente vascular cerebral [PI 0,68 (IC 95% 0,65; 0,71)], um ataque isquêmico transitório [PI 0,17 (IC 95% 0,16; 0,18)] e um tromboembolismo venoso [PI 0,17 (IC 95% 0,16; 0,18)]. Nenhum evento embólico sistêmico foi relatado.

**Conclusão:**

Em uma população idosa com várias comorbidades, recebendo edoxabana como tratamento de rotina para FA, as taxas de evento cardiovasculares sangramento maior foram baixas.

## Graphical abstract

**Figura Central:**
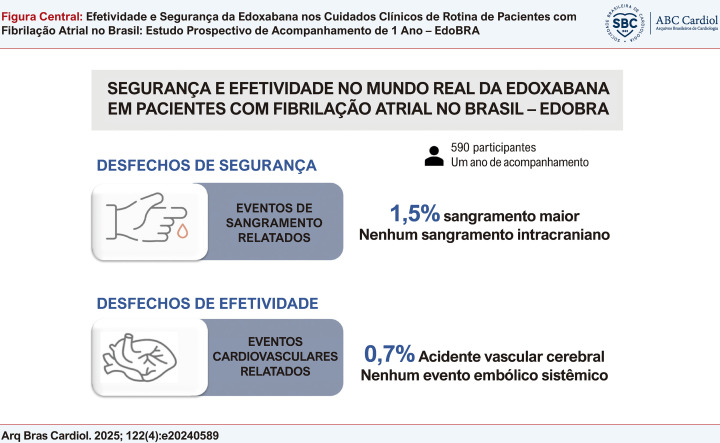
Efetividade e Segurança da Edoxabana nos Cuidados Clínicos de Rotina de Pacientes com Fibrilação Atrial no Brasil: Estudo Prospectivo de Acompanhamento de 1 Ano – EdoBRA

## Introdução

A fibrilação atrial (FA) é a arritmia cardíaca mais comum em adultos, principalmente em homens, e está associada a um aumento na morbidade e na mortalidade,^1^ representando uma carga significativa aos sistemas de saúde.^2,3^ Idade avançada (≥80 anos) é o fator de risco mais evidente para FA, seguida de comorbidades, tais como hipertensão, insuficiência cardíaca, obesidade, doença arterial coronariana, entre outros.^4^

O tratamento de primeira linha para prevenir o risco de acidente vascular cerebral (AVC) consiste em anticoagulantes orais. Os anticoagulantes orais não antagonistas da vitamina K (NOACs) surgiram como o tratamento de escolha dada a sua eficácia e o risco mais baixo de hemorragia intracraniana em comparação aos antagonistas de vitamina K (VKAs), que consiste na terapia padrão.^3,5,6^

A edoxabana é um NOAC com uma farmacocinética linear e previsível que inibe seletivamente o fator Xa. Seu uso foi aprovado na prática clínica em todo o mundo, incluindo o Brasil.^7^ A edoxabana vem se tornando cada vez mais a terapia padrão na prática clínica para prevenir eventos de AVC em pacientes com FA e para tratamento e prevenção de tromboembolismo venoso. Tipicamente, a edoxabana é prescrita para ser tomada na dose de 60mg uma vez ao dia. Contudo, para pacientes com condições clínicas específicas, recomenda-se um ajuste na dose para 30mg uma vez ao dia.^8^

No ensaio randomizado ENGAGE AF-TIMI 48, a edoxabana demonstrou eficácia e segurança em uma grande e diversificada população de pacientes, com taxas mais baixas de AVC hemorrágico, sangramentos maiores e morte por causas cardiovasculares em comparação ao VKA varfarina.^9^ Além disso, um estudo de mundo real (ETNA-AF-Europe)^10^ ilustrou a eficácia e a segurança da edoxabana (30 mg/dia e 60 mg/dia) no cuidado de rotina de pacientes europeus.^10^

Apesar de a segurança e a efetividade da edoxabana já estarem bem estabelecidas, informações a respeito de seu uso em abordagens clínicas são ainda escassas no Brasil. Para preencher essa lacuna no conhecimento, o presente estudo apresenta uma avaliação, durante um ano, da segurança e da eficácia da edoxabana em pacientes com FA atendidos em diferentes em regiões do Brasil.

## Métodos

### Delineamento do estudo e coleta de dados

Este estudo prospetivo, multicêntrico, observacional foi conduzido em 30 centros de pesquisa no Brasil sem uma agenda específica de visitas. Dados foram coletados dos prontuários médicos em quatro momentos ao longo de um ano, incluindo os pacientes que descontinuaram o uso de edoxabana. Detalhes do delineamento do estudo e dos métodos da coleta de dados foram descritos previamente.^11^

### População do estudo

**Figura 1:**
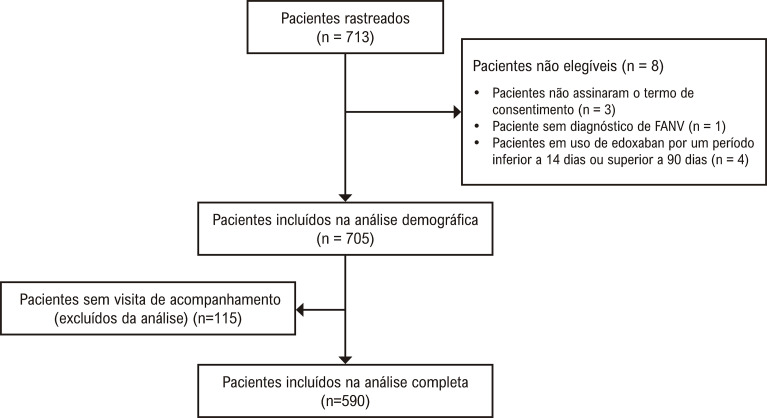
– Fluxograma do estudo.

O estudo recrutou 713 pacientes (Figura 1) entre setembro de 2019 e fevereiro de 2022 de vários centros de saúde. Os pacientes incluídos estavam em tratamento com edoxabana para Fibrilação Atrial Não-Valvar (FANV), tinham idade superior a 18 anos, estavam em uso de edoxabana por 14-90 dias antes de serem incluídos, e assinaram um termo de consentimento.

### Desfechos

Desfechos de segurança primária tiveram como foco eventos de sangramento, definido pela *International Society of Thrombosis and Haemostasis* (ISHT). Sangramento maior incluiu sangramento fatal ou sangramento sintomático em áreas críticas (como a região retroperitoneal e intracraniana). Sangramento não maior clinicamente relevante foi classificado como aqueles que não necessitaram de intervenção, hospitalização ou uma avaliação presencial.

Desfechos secundários para efetividade, incluíram eventos cardiovasculares como AVC (isquêmico ou hemorrágico), eventos embólicos sistêmicos (EES), ataque isquêmico transitório (AIT), tromboembolismo venoso (TEV), síndrome coronariana aguda (SCA) e eventos adversos cardiovasculares maiores – uma combinação de infarto do miocárdio (IM) não fatal, AVC não fatal, EES não fatais, e morte por causas cardiovasculares ou sangramento. Foram registradas todas as medidas de segurança e eventos adversos desde o recrutamento até o final do seguimento.

### Análise estatística

Foram realizadas análises descritivas de todos os dados coletados, e os resultados foram apresentados como frequências e porcentagens, excluindo os dados faltantes. Toda análise descritiva baseou-se nas informações disponíveis, e dados faltantes ou desconhecidos não foram considerados. As variáveis contínuas foram descritas em médias e desvios padrões (DPs) ou medianas e intervalos interquartis, conforme apropriado. Proporções de incidências (PIs) foram calculadas dividindo-se o número de pacientes com um evento pelo número total de pacientes (n=590), e expressas como porcentagens, com um Intervalo de Confiança (IC) de 95%. Curvas de Kaplan-Meier foram usadas para análise tempo vs. evento. As análises foram conduzidas seguindo as diretrizes STROBE e CONSORT.

## Resultados

### Características basais

O estudo incluiu 705 pacientes com FA tratados com edoxabana, sendo que 590 foram considerados para a análise completa. O tempo médio de acompanhamento foi de 11,3 meses. Os pacientes eram originários principalmente da região nordeste (42,0%), seguido da região sul (29,4%), sudeste (28,2%) e centro-oeste (0,3%).

No basal (início da edoxabana), a idade mediana foi 70 anos, e a maioria dos pacientes era do sexo masculino (60%). Os octogenários representaram 18% da população do estudo, com idade média de 84,96 anos (DP: 3,95 anos).

As etnias/raças mais comuns foram caucasiana (38,06%), hispânica/latina (30,97%), e afrodescendente (28,73%). A maioria dos pacientes (64%) foi tratada pelo sistema público de saúde (Tabela 1).

**Tabela 1:** – Características clínicas e demográficas basais

Variáveis	n=590
**Características demográficas**	
**Sexo, N (%)**	
Número (válido)	590
Homens	354 (60%)
Mulheres	236 (40%)
**Idade, anos**	
Número (válido)	590
Média ± DP	68,9 ± 12,6
mediana	70
**Etnia, N (%)**	
Número (válido)	536
Caucasiana	204 (38,06%)
Hispânica/Latina	166 (30,97%)
Afrodescendente	154 (28,73%)
Asiática	8 (1,49%)
Nativa	4 (0,75%)
**Seguro de saúde, N (%)**	
Número (válido)	508
Público	324 (64%)
Privado	184 (36%)
**Características clínicas**	
**Peso corporal, Kg**	
Número (válido)	507
média ± DP	77,48 ± 17,91
**IMC (Kg/m^2^), N (%)**	
Número (válido)	496
< 18,5	10 (2%)
18,5 - 24,9	140 (28%)
25 - 29,9	190 (38%)
> 30	156 (32%)
**Tipo de FA, N (%)**	
Número (válido)	543
Paroxística	215 (40%)
Persistente	93 (17%)
Persistente de longa duração	14 (2%)
Permanente	221 (41%)
**Sinais e sintomas, número de pacientes (%)**	
Número (válido)	525
Assintomático	382 (73%)
Sintomático	143 (27%)
**Pressão arterial, mmHg**	
Número (válido)	519
Sistólica, média ± DP	126,8 ± 19,31
Diastólica, média ± DP	76,39 ± 13,47
**Creatinina sérica, mg/dL**	
Número (válido)	492
média ± DP	1,46 ± 5,43
***Clearance* de creatinina, mL/min**	
Número (válido)	258
média ± DP	70,5 ± 35,15
**Escore CHA**_**2**_**DS**_**2**_**-VASc**	
Número (válido)	360
média ± DP	3,3 ± 1.6
**HAS-BLED, *Risk score***	
Número (válido)	269
média ± DP	1,8 ± 1,2
**Fragilidade, N (%)**	
Número (válido)	435
Sim	74 (17%)
Não	361 (83%)
**FEVE, N (%)**	
Número (válido)	229
≥ 40%	156 (68%)
< 40%	73 (32%)
**História de doença cardiovascular, N (%)**	
Número (válido)	590
Hipertensão	457 (78%)
Insuficiência cardíaca	164 (28%)
Infarto do miocárdio	66 (11%)
Doença coronariana	78 (13%)
AVC	52 (9%)
Doença arterial periférica	18 (3%)
AIT	16 (2,7%)
**Diabetes mellitus, N (%)**	
Número válido	573
Sim	172 (30%)
Não	401 (70%)
**Obesidade, N (%)**	
Número válido	588
Sim	147 (25%)
Não	441 (75%)
**Dislipidemia, N (%)**	
Número válido	581
Sim	244 (42%)
Não	337 (58%)
**DPOC, N (%)**	
Número válido	583
Sim	28 (4,8%)
Não	555 (95,2%)
**História de sangramento maior, N (%)**	
Número válido	579
Sim	11 (1,9%)
Não	568 (98,1%)

*^1^ Dados faltantes ou desconhecidos não foram considerados no cálculo das porcentagens. ^2^ Para história de doença cardiovascular, um paciente poderia ter mais de uma doença cardiovascular prévia. AIT: ataque isquêmico transitório; AVC: acidente vascular cerebral; DPOC: doença pulmonar obstrutiva crônica; FA: fibrilação atrial; FEVE: fração de ejeção ventricular esquerda; IMC: índice de massa corporal.*

O peso médio dos pacientes foi 77,5 Kg, e as comorbidades frequentes incluíram hipertensão (78%), dislipidemia (42%), e diabetes (30%).

Em quase três quartos dos pacientes, a FA foi assintomática. Os sintomas mais comuns relatados entre os pacientes sintomáticos foram palpitações (52%), dispneia (43%), e fadiga (32%). A FA permanente foi o tipo mais frequente (41%), seguido da FA paroxística. Os valores medianos do escore CHA2DS2-VASc e do escore HAS-BLED foram 3 e 2, respectivamente. De todos os pacientes, 17% se consideravam frágeis.

### Informações sobre a prescrição de Edoxabana

Mais da metade (51%) dos pacientes receberam edoxabana como o primeiro tratamento para FANV, e 23% receberam a medicação considerando a possibilidade de uma maior segurança ou eficácia. Uma menor dose (30mg/dia) foi dada a 30% dos pacientes (Tabela 2).

**Tabela 2:** – Informações sobre a prescrição de edoxabana

Variáveis	N = 590
**Dose de edoxabana, N (%)**	
Número (válido)	590
30mg uma vez ao dia	179 (30%)
60mg uma vez ao dia	411 (70%)
**Causa da prescrição, N (%)**	
Número (válido)	444
Primeiro tratamento da FANV com ACO	227 (51%)
Maior eficácia esperada	56 (13%)
Maior segurança esperada	45 (10%)
Alta variabilidade na resposta ao primeiro tratamento com ACO	35 (8%)
Falta de eficácia do primeiro tratamento com ACO	13 (3%)
Solicitação do paciente	13 (3%)
Maior adesão esperada	8 (2%)
Interação medicamentosa no primeiro tratamento	2 (0.5%)
Outros	45 (10%)
**Interrupção no tratamento com edoxabana, N (%)**	
Número de pacientes	117 (21%)
Número de interrupções	188 (13%)
Causa da interrupção*, Número de interrupções (%)	
Número (válido)	188
Evento adverso	42 (22%)
Solicitação do paciente	33 (18%)
Intervenção para FA	12 (6%)
Relacionada ao sistema de saúde	6 (3%)
Interação medicamentosa	1 (0,5%)
Outros	94 (50%)
**Reinício da edoxabana após a interrupção, N (%)**	
Número válido	117
Pacientes que retomaram o uso de edoxabana e ainda o continua	15 (13%)
Pacientes que interromperam o uso de edoxabana e não o retomou (decontinuou)	102 (87%)
**Duração do tratamento, meses**	
Número válido	590
Média ± DP	10,4 ± 3,8
Mediana	12,25
IQR (25-75)	5,58 (7,46 - 13,04)

*Causa de descontinuação relatada por um único paciente; um paciente podia apresentar mais de uma interrupção; FANV: fibrilação atrial não-valvar; ACO: anticoagulantes orais; IIQ: intervalo interquartil; FA: fibrilação atrial.

O tratamento foi interrompido em 21% dos pacientes, com 188 interrupções registradas ao longo do estudo. As razões incluíram eventos adversos (22%), vontade do paciente (18%), intervenção para a FA (6%), questões relacionadas ao serviço de saúde (3%), interações medicamentosas (0,5%), e outras razões (50%). Entre essas, a mais relatada foram questões financeiras (10%; N=18).

Após a descontinuação do uso da edoxabana, 13% reiniciaram seu uso, e 87% dos pacientes a interromperam permanentemente (Tabela 2). Após o acompanhamento de um ano, 83% dos pacientes ainda usavam edoxabana.

### Desfechos de segurança

**Figura 2:**
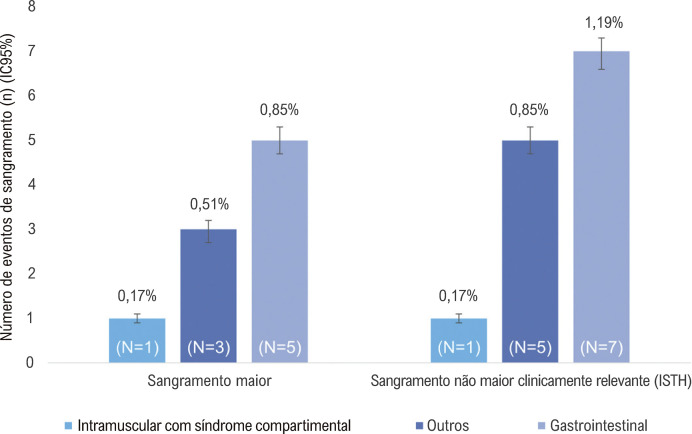
– Evento de sangramento de acordo com o International Society on Thrombosis and Haemostasis (sangramento maior e sangramento não maior clinicamente relevante); PI: proporção de incidência; IC: intervalo de confiança. Número de eventos de sangramento (n) (IC95%)

A Figura 2 apresenta a ocorrência de sangramento maior e eventos de sangramento não maiores clinicamente importantes, segundo o *International Society on Thrombosis and Haemostasis* (ISTH), durante um ano de seguimento. Um total de 13 eventos primários de segurança foram registrados durante o período do estudo; nove eventos de sangramento maior foram relatados, dos quais cinco ocorreram no trato gastrointestinal [PI 0,85 [IC95% =0,82; 0,88]), um consistiu em síndrome compartimental (PI 0,17 [IC95% =0,16; 0,18]) e três eventos foram classificados como “outros” (PI 0,51 [IC95% = 0,48; 0,53]). Em relação às causas dos eventos de sangramento maior, seis foram consideradas espontâneas [PI 1,02 (IC 95%= 0,98; 1,05)] e três eventos tiveram causa relatada como desconhecida. Entre os eventos de sangramento maior, 2 (PI 0,34 [IC95% =0,32; 0,36]) deles levaram a um desfecho fatal.

Entre os quatro casos de sangramento não maiores clinicamente importantes, dois foram sangramento gastrointestinal (PI 0,34 [IC95% =0,32; 0,36]), e dois foram classificados como outros (PI 0,34 [IC95% =0,32; 0,36]). Todos os quatro casos [PI 0,68 [IC95% =0,65; 0,71]) foram relatados como espontâneos.

Considerando todos os casos de sangramentos maiores e não maiores (n=5), quatro foram relacionados ao sistema geniturinário e um foi registrado como sangramento nasal (dados não apresentados). Não foi relatado sangramento intracraniano durante o estudo.

Entre os casos de sangramento maior e sangramento não maior clinicamente relevante, nove foram relacionados à edoxabana (Tabela 3). Desses, sete (PI 1,19 [IC95% 1,15; 1,22]) foram eventos de sangramento maior e dois (PI 0,34 [IC95% 0,32;0,36]) foram sangramentos não maiores clinicamente importantes. Considerando o tipo de sangramento, a maioria foi sangramento gastrointestinal (N=5; PI 0,85 [IC95% 0,82; 0,88)].

**Tabela 3:** – Eventos de sangramento relacionados à edoxabana

	Eventos (n)	Pacientes (n)	PI (IC95%)
**Sangramento (ISTH)**			
Sangramento maior	7	7	1,19 (1,15;1,22)
CRNMB	2	2	0,34 (0,32;0,36)
**Tipo de sangramento**			
Gastrointestinal	5	5	0,85 (0,82;0,88)
Síndrome compartimental	1	1	0,17 (0,16;0,18)
Outros	3	3	0,51 (0,48;0,53)

*PI: Proporção de Incidência; ISTH: International Society on Thrombosis and Haemostasis.*

Foram relatados 12 eventos de sangramento menor: dois eventos gastrointestinais (PI 0,34), um intraocular (PI 0,17), e nove outros (Tabela S1). Entre outros tipos de eventos de sangramento menor, o mais comum foi epistaxe (n=3) (dados não apresentados). Onze eventos (PI 1,86) tiveram causas espontâneas.

### Desfechos de efetividade

**Figura 3:**
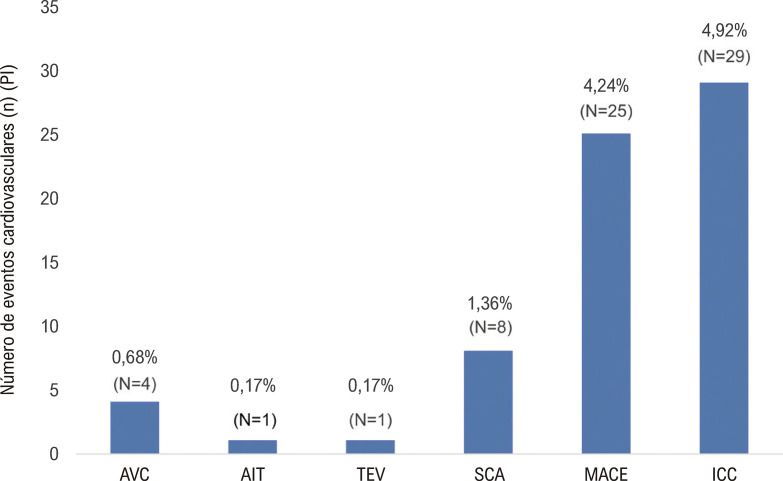
– Eventos cardiovasculares relatados ao longo do estudo; AVC: acidente vascular cerebral; AIT: Ataque Isquêmico Transitório; TEV: tromboembolismo venoso SCA: síndrome coronariana aguda; ICC: insuficiência cardíaca crônica.

Em relação aos desfechos de efetividade, a Figura 3 apresenta a incidência de eventos cardiovasculares entre os pacientes com FA. A avaliação da efetividade mostrou que, no geral, 112 (23,9%) pacientes apresentaram 141 eventos cardiovasculares [PI 23,9 [IC95% 23,76; 24,04]). Quatro eventos de AVC [PI 0,68 [IC95% 0,65; 0,71)] foram registrados durante o estudo – três [PI 0,51 (IC 95% 0,48; 0,53)] AVC isquêmico e um foi considerado AVC de causa desconhecida [PI 0,17 (IC 95% 0,16; 0,18]) (dados não apresentados). A PC de SCA [N=8; PI 1,36 (IC95% 1,32; 1,39)], AIT [N=1; PI 0,17 (IC 95% (0,16;0.18]) e TEV (N=1; PI 0,17 [IC 95% (0,16; 0,18]) foi mais baixa em comparação a outros eventos cardiovasculares, representando menos de 2% dos pacientes. Nenhum paciente apresentou EES.

Insuficiência cardíaca congestiva [N=29; PI 4,92 [IC 95% 4,84; 4,99]) e eventos adversos cardiovasculares maiores [(N=25; PI 4,24 (IC95% 4,17; 4,3)] apresentaram a maior PI, com aproximadamente 5% dos pacientes apresentando pelo menos um dos eventos.

Em relação aos eventos cardiovasculares relacionados à edoxabana, foi registrado um AIT [PI 0,17 IC95% CI (0,16; 0,18)] ao longo do estudo.

No geral, 107 pacientes apresentaram 130 eventos classificados como eventos adversos sérios [PI 22,03 (IC95% 21,9; 22,17)]. Entre esses, 58 eventos resultaram em óbito [PI 9,83 (IC95% 9,73; 9,93)], mas a maioria dos eventos (N= 75) resultaram em recuperação (PI 12.71 [95% CI 12,6;12,82]).

**Figura 4:**
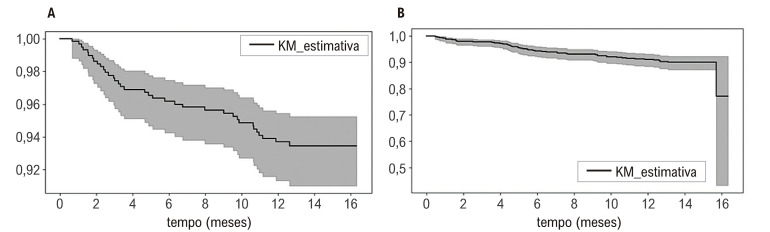
– Curvas de Kaplan-Meier do tempo (meses) entre o basal e o evento cardiovascular (A) e do tempo entre o basal e óbito (B).

Quanto aos óbitos que ocorreram ao longo do estudo, dois foram relacionados a eventos de sangramento maior e um a AIT (dados não apresentados). Apesar do número limitado de eventos entre os participantes, o que dificulta uma estimativa precisa do tempo para o evento, a Figura 4 apresenta as análises de Kaplan-Meier do tempo (em meses) entre o início do uso da edoxabana e a ocorrência de quaisquer eventos cardiovasculares (A). Ainda, a figura ilustra a estimativa Kaplan-Meier do basal até o óbito relacionado a eventos cardiovasculares (B).

## Discussão

Este é um estudo prospectivo, não intervencionista, sobre um NOAC, que apresenta dados sobre desfechos de pacientes tratados rotineiramente com edoxabana em centros de saúde no Brasil, e participantes do estudo EdoBRA. Os resultados reforçam a segurança e a eficácia da edoxabana, principalmente em termos de sangramento, e eventos cardiovasculares e tromboembólico na prática clínica.

O estudo incluiu 705 participantes de 30 locais do Brasil. Entre eles, 590 estiveram presentes em pelo menos uma visita de acompanhamento, ou relataram um evento, tornando-os elegíveis para a inclusão na análise completa. Embora o estudo tenha sido realizado entre 2019 e 2023, um período que englobou a pandemia de COVID-19, a média de tempo de seguimento está de acordo com o período médio de um estudo similar conduzido em outro período.^10^

Características demográficas da população do estudo EdoBRA estão de acordo com as apresentadas em estudos anteriores.^10,12,13^ Ainda, os resultados do EdoBRA ilustram que os participantes eram em sua maioria idosos no início do uso da edoxabana, com uma idade média de 68,9 anos, a qual é um pouco inferior à comparada à de populações similares como no ETNA-AF (73,6 ± 9,46)^10^ e no ENGAGE AF-TIMI-48 (71,4 ± 9,7).^9^ Além disso, o presente estudo demonstrou que 18% dos pacientes com idade igual ou acima de 80 anos, que é um pouco mais alta que os dados da pesquisa nacional de saúde conduzida em 2013 pelo Ministério da Saúde que mostrou que os octogenários representavam 13,6% da população (≥ 60 anos). Isso reflete uma tendência global de uma população em envelhecimento. O Brasil possui uma das maiores populações em envelhecimento no mundo. De acordo com estimativas do censo do Instituto Brasileiro de Geografia e Estatística de 2022, há 4,6 milhões de brasileiros com idade igual ou maior que 80 anos, representando cerca de 2,27% da população total.^14^

Por outro lado, os participantes do EdoBRA em geral apresentaram proporções mais altas de comorbidades, principalmente insuficiência cardíaca e AVC em comparação ao estudo ETNA-AF.^10^ A hipertensão, um fator de risco conhecido e geralmente comum entre os pacientes com FA foi a principal comorbidade observada na população do estudo, o que está de acordo com os achados na literatura.^3,9,10^ O processo do envelhecimento e a presença de comorbidades estão associados com um risco aumentado de eventos tromboembólicos e de sangramento em pacientes com FA.^15^

Considerando as características da FA, 40% dos pacientes com EdoBRA apresentaram o tipo avançado de FA (permanente) no basal. Apesar de esse resultado parecer mais alto que a porcentagem (22%) relatada no estudo ETNA,^10^ ele está de acordo com os resultados relatados no estudo ENGAGE AF-TIMI-48, que demonstrou que pacientes mais velhos e pacientes com FA permanente, fração de ejeção ventricular esquerda mais baixa, entre outras características, eram mais propensas à morte súbita ou à apresentarem insuficiência cardíaca.^9^ Ainda, fatores relacionados ao estilo de vida, tais como índice de massa corporal elevado e uma história de diabetes, estão associados com progressão de FA. Esses fatores podem ajudar a explicar a elevada porcentagem de FA permanente observada no basal nos pacientes do EdoBRA.^16^

Quase três quartos (73%) dos pacientes do estudo EdoBRA apresentaram FA assintomática no basal. A prevalência da apresentação assintomática entre os pacientes com FA ainda não foi estabelecida; alguns estudos sugerem porcentagens entre 10% e 40%, dependendo das características da população.^17^ Quanto aos sintomas de FA, os mais frequentemente relatados – palpitação, dispneia e fadiga – são os sintomas mais comuns relacionados à FA.^18^ Ainda, os escores CHA2DS2-VASc e HAS-BLED, que avaliam o risco de AVC e sangramento, respectivamente, demonstraram escores medianos de 3,3 e 1,8, no basal. Esses escores mantiveram-se consistentes ao longo de todo o estudo (dados não apresentados). Os valores do escore CHA2DS2-VASc foram similares aos resultados do estudo ETNA-AF; no entanto, foram mais baixos em comparação à população não asiática do estudo ENGAGE AF-TIMI-48 (4,3 ± 1,41).^9^ Considerando o escore de risco HAS-BLED, os valores estão de acordo com o estudo ENGAGE AF-TIMI-48, porém mais baixos que o da população do estudo ETNA-AF-Europe (2,5 ± 1,10).^10^ Esses resultados indicam um perfil de alto risco para eventos tromboembólicos e um risco moderado de sangramento na população do estudo.^19,20^

As características da prescrição de edoxabana indicaram que aproximadamente 30% dos pacientes receberam prescrição de 30mg, uma vez ao dia. Cerca de metade dos pacientes estavam recebendo tratamento com anticoagulantes orais para FANV pela primeira vez. Em relação à dose de edoxabana. Os critérios para redução da dose (disfunção renal, peso corporal mais baixo, e tratamento concomitante com inibidor de glicoproteína P) são marcadores para pacientes com alto risco de eventos tromboembólicos e de sangramento.^21^ Embora os números corroborem de alguma forma que pacientes do estudo EdoBRA apresentaram proporções maiores de características de risco, a dose prescrita estava de acordo com a literatura, uma vez que 30% dos pacientes do estudo EdoBRA estavam tomando 30mg.^9,10^ A dosagem dos NOACs continuam um desafio na prática clínica.^21^

A persistência do tratamento continuou elevado ao longo do estudo, com uma taxa de descontinuação de 21%. Esses resultados são mais altos que os recentemente descritos no estudo ETNA-AF^10^ que demonstrou uma taxa de descontinuação menor que 10%. No entanto, os resultados parecem ser concordantes com outros registros que demonstraram taxas de descontinuação superiores a 15% durante o primeiro ano de seguimento.^22,23^

Entre os motivos para a descontinuação da edoxabana, 18% estavam relacionados à solicitação do paciente e 10% relacionados a questões financeiras. A maioria dos pacientes incluídos no presente estudo dependiam de serviços públicos de saúde, caracterizando uma população de baixa renda.^24^ Apesar das evidências de que as taxas de descontinuação de NOAC sejam mais baixas em comparação às observadas com VKAs, o custo do tratamento ainda é uma questão relevante em relação à descontinuação de NOAC.^21^

O estudo EdoBRA mostrou baixas taxas de AVC e sangramentos. Esses resultados reforçam dados da segurança e da efetividade de um ensaio clínico randomizado com NOACs.^25^ Os resultados do EdoBRA corroboram os achados do primeiro estudo internacional prospectivo com pacientes tratados com rivaroxabana (registro XANTUS),^23^ e do ETNA-AF-Europe, um estudo pós-autorização.^10^ Resultados similares também foram relatados em estudos retrospectivos.^26,27^ Resultados de um grande estudo de dois anos de seguimento também corroboram esses achados, mostrando ausência de diferenças significativas entre pacientes tomando ambas as doses e edoxabana.^28^ Resultados similares quanto à redução de eventos de sangramento pela edoxabana foram encontrados em participantes do leste asiático em comparação à varfarina. O relatório de um ano do registro de longo prazo EORP^29^ também demonstrou baixas taxas de AVC e sangramento em pacientes tratados com NOACs.^29^

Considerando todos os eventos ocorridos durante o estudo, a maioria resultou na recuperação do paciente, e 58 na morte do paciente, representando 9,8% da população do estudo. As taxas de mortalidade por todas as causas em pacientes usando edoxabana foram baixas no estudo ETNA-AF, com uma taxa de 1,6% ao ano.^10^ A coorte da América Latina no estudo ENGAGE AF-TIMI-48 em uso de edoxabana apresentou uma taxa de mortalidade anual superior a 6%.^9^ Apesar da taxa mais alta de mortalidade no estudo EdoBRA, somente três casos se relacionaram a sangramento ou a eventos cardiovasculares (dados não apresentados), o que corrobora os resultados na literatura de pacientes usando edoxabana.^9,10,30^

Foi demonstrado que a edoxabana reduz a mortalidade entre pacientes com FA, principalmente por diminuir a ocorrência de sangramentos.^30^ A pandemia da COVID-19 é um fator importante quando se analisa esses resultados, não só pelo volume de mortes cumulativas durante o período,^31^ como também por seu impacto sobre o acompanhamento da FA. Por exemplo, atrasos na prescrição possivelmente foram causados por políticas de restrições de mobilidade social. Durante a pandemia, os pacientes também relataram dificuldades em agendar consultas ambulatoriais, com um aumento nas consultas médicas virtuais.^32^

Os pontos fortes do presente estudo incluem seu delineamento prospectivo e a ausência de restrição à participação no estudo como uma característica de estudos observacionais, corroborando a possibilidade de generalizar os dados de pesquisa. Ainda, embora o estudo tenha sido conduzido durante a pandemia da COVID-19, o tempo mediano de acompanhamento é consistente com o de estudos conduzidos em outros períodos.

Apesar de as taxas de acompanhamento no presente estudo sejam concordantes com os dados mostrados na literatura, em estudos de mundo real, a probabilidade de se obter informações faltantes do paciente é menor, uma vez que, no geral, diferentemente de ensaios clínicos, as consultas dos pacientes não são agendadas no local da pesquisa. Ainda, o estudo foi conduzido durante a pandemia da COVID-19, o que impôs desafios importantes aos pacientes quanto ao agendamento de consultas e exames.

Outra limitação do estudo é a inevitável comparação dos resultados encontrados neste estudo observacional com resultados de ensaios clínicos. Além disso, pelo fato de a maioria dos pacientes do presente estudo serem originários da região nordeste de Brasil, é necessário cautela em se extrapolar os resultados à toda população no país, devido às diferenças socioeconômicas e culturais entre as regiões.

## Conclusão

O estudo EdoBRA relatou baixas taxas de eventos cardiovasculares e sangramentos durante o período de um ano de acompanhamento, apesar da população idosa e acúmulo de comorbidades. Os resultados reforçam a efetividade e a segurança da edoxabana no tratamento de rotina de pacientes com FANV no Brasil.

## References

[1] Brundel BJJM, Ai X, Hills MT, Kuipers MF, Lip GYH, Groot NMS (2022). Atrial Fibrillation. Nat Rev Dis Primers.

[2] Benjamin EJ, Muntner P, Alonso A, Bittencourt MS, Callaway CW, Carson AP (2019). Heart Disease and Stroke Statistics-2019 Update: A Report from the American Heart Association. Circulation.

[3] Hindricks G, Potpara T, Dagres N, Arbelo E, Bax JJ, Blomström-Lundqvist C (2021). 2020 ESC Guidelines for the Diagnosis and Management of Atrial Fibrillation Developed in Collaboration with the European Association for Cardio-Thoracic Surgery (EACTS): The Task Force for the Diagnosis and Management of Atrial Fibrillation of the European Society of Cardiology (ESC) Developed with the Special Contribution of the European Heart Rhythm Association (EHRA) of the ESC. Eur Heart J.

[4] Bizhanov KA, Abzaliyev KB, Baimbetov AK, Sarsenbayeva AB, Lyan E (2023). Atrial Fibrillation: Epidemiology, Pathophysiology, and Clinical Complications (Literature Review). J Cardiovasc Electrophysiol.

[5] January CT, Wann LS, Alpert JS, Calkins H, Cigarroa JE, Cleveland JC (2014). 2014 AHA/ACC/HRS Guideline for the Management of Patients with Atrial Fibrillation: Executive Summary: A Report of the American College of Cardiology/American Heart Association Task Force on Practice Guidelines and the Heart Rhythm Society. Circulation.

[6] Lip GYH, Banerjee A, Boriani G, Chiang CE, Fargo R, Freedman B (2018). Antithrombotic Therapy for Atrial Fibrillation: CHEST Guideline and Expert Panel Report. Chest.

[7] Agência Nacional de Vigilância Sanitária (2018). Lixiana (Edoxabana) - Parecer Público de Avaliação de Medicamento.

[8] Parasrampuria DA, Truitt KE (2016). Pharmacokinetics and Pharmacodynamics of Edoxaban, a Non-Vitamin K Antagonist Oral Anticoagulant that Inhibits Clotting Factor Xa. Clin Pharmacokinet.

[9] Corbalán R, Nicolau JC, López-Sendon J, Garcia-Castillo A, Botero R, Sotomora G (2018). Edoxaban versus Warfarin in Latin American Patients with Atrial Fibrillation: The ENGAGE AF-TIMI 48 Trial. J Am Coll Cardiol.

[10] Groot JR, Weiss TW, Kelly P, Monteiro P, Deharo JC, Asmundis C (2021). Edoxaban for Stroke Prevention in Atrial Fibrillation in Routine Clinical Care: 1-Year Follow-Up of the Prospective Observational ETNA-AF-Europe Study. Eur Heart J Cardiovasc Pharmacother.

[11] Précoma DB, Silva RPD, Nakamoto A, Omar VM, Lopes D, Saraiva JFK (2024). Study Design of a Brazilian Observational Study of Edoxaban in Patients with Atrial Fibrillation (EdoBRA). Arq Bras Cardiol.

[12] Ruigómez A, Vora P, Balabanova Y, Brobert G, Roberts L, Fatoba S (2019). Discontinuation of Non-Vitamin K Antagonist Oral Anticoagulants in Patients with Non-Valvular Atrial Fibrillation: A Population-Based Cohort Study Using Primary Care Data from The Health Improvement Network in the UK. BMJ Open.

[13] Berger JS, Laliberté F, Kharat A, Lejeune D, Moore KT, Jung Y (2021). Real-World Effectiveness and Safety of Rivaroxaban versus Warfarin Among Non-Valvular Atrial Fibrillation Patients with Obesity in a US Population. Curr Med Res Opin.

[14] Instituto Brasileiro de Geografia e Estatística (2022). Censo Demográfico 2022.

[15] Sabbag A, Yao X, Siontis KC, Noseworthy PA (2018). Anticoagulation for Stroke Prevention in Older Adults with Atrial Fibrillation and Comorbidity: Current Evidence and Treatment Challenges. Korean Circ J.

[16] Blum S, Aeschbacher S, Meyre P, Zwimpfer L, Reichlin T, Beer JH (2019). Incidence and Predictors of Atrial Fibrillation Progression. J Am Heart Assoc.

[17] Ballatore A, Matta M, Saglietto A, Desalvo P, Bocchino PP, Gaita F (2019). Subclinical and Asymptomatic Atrial Fibrillation: Current Evidence and Unsolved Questions in Clinical Practice. Medicina (Kaunas).

[18] Michaud GF, Stevenson WG (2021). Atrial Fibrillation. N Engl J Med.

[19] Yi JE, Lee YS, Choi EK, Cha MJ, Kim TH, Park JK (2018). CHA2DS2-VASc Score Predicts Exercise Intolerance in Young and Middle-Aged Male Patients with Asymptomatic Atrial Fibrillation. Sci Rep.

[20] Caterina R, Kim YH, Koretsune Y, Wang CC, Yamashita T, Chen C (2021). Safety and Effectiveness of Edoxaban in Atrial Fibrillation Patients in Routine Clinical Practice: One-Year Follow-Up from the Global Noninterventional ETNA-AF Program. J Clin Med.

[21] Steffel J, Collins R, Antz M, Cornu P, Desteghe L, Haeusler KG (2021). 2021 European Heart Rhythm Association Practical Guide on the Use of Non-Vitamin K Antagonist Oral Anticoagulants in Patients with Atrial Fibrillation. Europace.

[22] Beyer-Westendorf J, Förster K, Ebertz F, Gelbricht V, Schreier T, Göbelt M (2015). Drug Persistence with Rivaroxaban Therapy in Atrial Fibrillation Patients-Results from the Dresden Non-Interventional Oral Anticoagulation Registry. Europace.

[23] Camm AJ, Amarenco P, Haas S, Hess S, Kirchhof P, Kuhls S (2016). XANTUS: a Real-World, Prospective, Observational Study of Patients Treated with Rivaroxaban for Stroke Prevention in Atrial Fibrillation. Eur Heart J.

[24] Coube M, Nikoloski Z, Mrejen M, Mossialos E (2023). Inequalities in Unmet Need for Health Care Services and Medications in Brazil: A Decomposition Analysis. Lancet Reg Health Am.

[25] Eisen A, Ruff CT, Braunwald E, Nordio F, Corbalán R, Dalby A (2016). Sudden Cardiac Death in Patients with Atrial Fibrillation: Insights from the ENGAGE AF-TIMI 48 Trial. J Am Heart Assoc.

[26] Denas G, Gennaro N, Ferroni E, Fedeli U, Saugo M, Zoppellaro G (2017). Effectiveness and Safety of Oral Anticoagulation with Non-Vitamin K Antagonists Compared to Well-Managed Vitamin K Antagonists in Naïve Patients with Non-Valvular Atrial Fibrillation: Propensity Score Matched Cohort Study. Int J Cardiol.

[27] Lip GYH, Keshishian A, Li X, Hamilton M, Masseria C, Gupta K (2018). Effectiveness and Safety of Oral Anticoagulants Among Nonvalvular Atrial Fibrillation Patients. Stroke.

[28] Kirchhof P, Pecen L, Bakhai A, Asmundis C, Groot JR, Deharo JC (2022). Edoxaban for Stroke Prevention in Atrial Fibrillation and Age-Adjusted Predictors of Clinical Outcomes in Routine Clinical Care. Eur Heart J Cardiovasc Pharmacother.

[29] Boriani G, Proietti M, Laroche C, Fauchier L, Marin F, Nabauer M (2019). Association between Antithrombotic Treatment and Outcomes at 1-Year Follow-Up in Patients with Atrial Fibrillation: The EORP-AF General Long-Term Registry. Europace.

[30] Giugliano RP, Ruff CT, Wiviott SD, Nordio F, Murphy SA, Kappelhof JA (2016). Mortality in Patients with Atrial Fibrillation Randomized to Edoxaban or Warfarin: Insights from the ENGAGE AF-TIMI 48 Trial. Am J Med.

[31] Szwarcwald CL, Boccolini CS, Almeida WS, Soares AM, Malta DC (2022). COVID-19 Mortality in Brazil, 2020-21: Consequences of the Pandemic Inadequate Management. Arch Public Health.

[32] Goulart AC, Varella AC, Gooden TE, Lip GYH, Jolly K, Thomas GN (2023). Identifying and Understanding the Care Pathway of Patients with Atrial Fibrillation in Brazil and the Impact of the COVID-19 Pandemic: A Mixed-Methods Study. PLoS One.

