# Supplementary material for: Efetividade e Segurança da Edoxabana nos Cuidados Clínicos de Rotina de Pacientes com Fibrilação Atrial no Brasil: Estudo Prospectivo de Acompanhamento de 1 Ano – EdoBRA
Source: Arq Bras Cardiol. 2025 Apr 2;122(4):e20240589. [Article in Portuguese] doi: 10.36660/abc.20240589 (PMC12058141; doi:10.36660/abc.20240589)
Supplement: Translation

# Translation: Effectiveness and Safety of Edoxaban in the Routine Clinical Care of Atrial Fibrillation Patients in Brazil: Prospective 1-Year Follow-Up Study – EdoBRA

**DOI:** 10.36660/abc.20240589i

**Authors:** Dalton Bertolim Precoma, Rafael Paletta da Silva, Luiz Carlos Santana Passos, Conrado Roberto Hoffman, Fábio Serra Silveira, José Tarcísio Medeiros de Vasconcelos, Sérgio Luiz Zimmermann, Artur Haddad Herdy, Alessandra Ritter, Daniely Freitas-Alves, José Francisco Kerr Saraiva

**Affiliations:** 1 Brazil Sociedade Hospitalar Angelina Caron, Campina Grande do Sul, PR – Brazil; 2 Brazil Daiichi Sankyo Brasil Farmaceutica LTDA, São Paulo, SP – Brazil; 3 Brazil Universidade Federal da Bahia, Salvador, BA – Brazil; 4 Brazil Hospital Regional Hans Dieter Schmidt, Joinville, SC – Brazil; 5 Brazil JMF Clínica do Coração, Aracaju, SE – Brazil; 6 Brazil Real e Benemérita Associação Portuguesa de Beneficência, São Paulo, SP – Brazil; 7 Brazil Instituto do Coração do Hospital das Clínicas da Faculdade de Medicina da Universidade de São Paulo, São Paulo, SP – Brazil; 8 Brazil Universidade Regional de Blumenau – Clínica Procardio, Blumenau, SC – Brazil; 9 Brazil Instituto de Cardiologia de Santa Catarina, São José, SC – Brazil; 10 Brazil IQVIA Brasil, São Paulo, SP – Brazil; 11 Brazil Pontifícia Universidade Católica de Campinas, Campinas, SP – Brazil

**Keywords:** Anticoagulants, Atrial Fibrillation, Hemorrhage, Stroke

## Abstract

**Background:**

Edoxaban, an orally administered anticoagulant, has been shown to be safe and effective in preventing stroke in atrial fibrillation (AF) patients. Given its widespread use since approval, evaluating edoxaban’s real-world performance in the Brazilian clinical context is crucial.

**Objective:**

The study aimed to report the one-year safety and effectiveness of edoxaban in AF patients in Brazil.

**Methods:**

EdoBRA is a multi-center, prospective, observational investigation conducted across 30 Brazilian research sites. Bleeding events were considered as safety measures and cardiovascular events were considered for effectiveness measures. Descriptive analyses were performed. Kaplan-Meier curves were generated for time-to-event analysis and a 95% confidence interval was used as appropriate.

**Results:**

Among the 705 enrolled participants, 590 were included in the analysis for having at least one follow-up or one reported event. Mean (±SD) CHA2DS2-VASc risk score was 3 (3.3 ± 1.6) and the mean HAS-BLED risk score was 2 (1.8 ± 1.2). During the one-year follow-up period, nine major bleedings events were reported, including five cases of gastrointestinal bleeding (IP 0.85 [95% CI =0.82; 0.88]). Among the cardiovascular events recorded (N = 68), there were four stroke events (IP 0.68 [CI 95% 0.65;0.71]), one transient ischemic attack (IP 0.17 [CI 95% (0.16;0.18]) and 1 event was Venous Thromboembolic Events (IP 0.17 [CI 95% (0.16;0.18]). No systemic embolic event was exhibited by any patient.

**Conclusion:**

In an elderly population with several comorbidities routinely treated with edoxaban for AF, the rates of cardiovascular event and major bleeding were low.

## Introduction

Atrial fibrillation (AF) is the most common cardiac arrhythmia in adults, especially men and is associated with an increase in patients’ morbidity and mortality,^1^ representing a significant burden to health systems.^2,3^ Advanced age (≥ 80 years of age) is the most prominent risk factor for AF followed by comorbidities such as high blood pressure, heart failure, obesity, coronary artery disease, among others.^4^

First-line treatment to prevent stroke risk in patients with AF consists of oral anticoagulants. Non-vitamin K oral anticoagulants (NOACs) have emerged as the preferred choice due to their efficacy and lower risk of intracranial hemorrhage compared to vitamin K antagonists (VKAs), the standard therapy.^3,5,6^

Edoxaban is a NOAC with a linear and predictable pharmacokinetics that selectively inhibits coagulation factor Xa. It has been approved for use in clinical practice all over the world, including in Brazil.^7^ It is increasingly becoming the standard of care in clinical practice to prevent stroke events in patients with AF and for treatment and prevention of venous thromboembolism. Edoxaban is typically prescribed once daily at a 60mg dose However, for patients with specified clinical conditions, a dose adjustment to 30mg once daily is recommended.^8^

In the randomized ENGAGE AF-TIMI 48 trial, edoxaban demonstrated both efficacy and safety across a large and diverse patient population, exhibiting lower rates of hemorrhagic stroke, major bleedings and death from cardiovascular causes when compared to the VKA warfarin.^9^ Additionally, a real-world evidence study (ETNA-AF-Europe)^10^ illustrated the effectiveness and safety of edoxaban 30 mg/day and 60 mg/day regimens in routine care in Europe.^10^

Although the safety and effectiveness of edoxaban have been well stablished, information regarding its use in clinical approaches is scarce in Brazil. To bridge the gap in knowledge, the present study presents an evaluation of the safety and effectiveness of edoxaban in a regular clinical care setting in patients with AF during 1 year of follow-up in different regions in Brazil.

## Methods

### Study design and data collection

This observational, multi-center, prospective study was conducted at 30 Brazilian research sites without a specific visit schedule. Data were collected from medical records at four time points over a one-year follow-up period, including patients who discontinued edoxaban. Detailed study design and data collection methods were previously described.^11^

### Study population

The study recruited 713 patients (Figure 1) from September 2019 to February 2022 from various care settings. Patients were included if they were assigned to edoxaban for nonvalvular atrial fibrillation (NVAF), were over 18 years old, had been on edoxaban for 14 to 90 days before enrollment, and provided written informed consent.

**Figure 1:** – Study flowchart.

### Outcomes

Primary safety outcomes focused on bleeding events, defined by the International Society of Thrombosis and Haemostasis (ISHT). Major bleeding included fatal bleeding or symptomatic bleeding in critical areas (e.g., retroperitoneal, intracranial). Clinically relevant non-major bleeding (CRNMB) was classified as the ones that required medical intervention, hospitalization, or a face-to-face evaluation.

Secondary outcomes for effectiveness included cardiovascular events like strokes (ischemic and hemorrhagic), systemic embolic events (SEE), transient ischemic attack (TIA), venous thromboembolism (VTE), acute coronary syndrome (ACS), and MACE (major adverse cardiovascular events) – e a composite of non-fatal myocardial infarction (MI), non-fatal stroke, non-fatal SEE, and death due to cardiovascular causes or bleeding. All safety measures and adverse events from enrollment to final follow-up were recorded.

### Statistical analysis

Descriptive analysis of all data collected was conducted, and results were shown as absolute frequencies and percentages, excluding missing data. All descriptive statistics were based on the available information, missing and unknown data were not considered. Continuous variables were described as means and standard deviations (SD) or medians and interquartile ranges, as appropriate. Incidence proportions (IPs) were calculated by dividing the number of patients with an event by the total number of patients (n = 590), and expressed as percentages, with a 95% confidence interval (CI). Kaplan-Meier curves were used for time-to-event analysis. The analysis followed STROBE and CONSORT guidelines.

## Results

### Baseline characteristics

The study included 705 patients with AF assigned to edoxaban, with 590 considered for full analysis. The mean follow-up was 11.3 months. Patients were mainly from northeast Brazil (42.0%), followed by south (29.4%), southeast (28.2%), and mid-west (0.3%).

At baseline (edoxaban initiation), the median age was 70 years, with a male predominance (60%). Octogenarians represented 18% of the study population with a mean age of 84.96 (SD: 3.95 years).

The most common races/ethnicities were Caucasian (38.06%), Hispanic/Latino (30.97%), and Black or Afro-descendent (28.73%). Most patients (64%) were treated through the public healthcare system (Table 1).

**Table 1:** – Baseline demographic and clinical features

Variables	n=590
**Demographic features**	
**Gender, N (%)**	
Valid numbers	590
Male	354 (60%)
Female	236 (40%)
**Age, years**	
Valid number	590
mean ± SD	68.9 ± 12.6
median	70
**Ethnicity, N (%)**	
Valid number	536
Caucasian	204 (38.06%)
Hispanic/Latino	166 (30.97%)
Black or Afro-descendent	154 (28.73%)
Asian	8 (1.49%)
Native	4 (0.75%)
**Healthcare Insurance, N (%)**	
Valid number	508
Public	324 (64%)
Private	184 (36%)
**Clinical Features**	
**Body weight, Kg**	
Valid number	507
mean ± SD	77.48 ± 17.91
**BMI (Kg/m^2^), N (%)**	
Valid n	496
< 18.5	10 (2%)
18.5 - 24.9	140 (28%)
25 - 29.9	190 (38%)
> 30	156 (32%)
**AF type, N (%)**	
Valid n	543
Paroxysmal	215 (40%)
Persistent	93 (17%)
Long standing persistent	14 (2%)
Permanent	221 (41%)
**Signs and symptoms, N of patients (%)**	
Valid n	525
Asymptomatic	382 (73%)
Symptomatic	143 (27%)
**Blood pressure, mmHg**	
Valid n	519
Systolic, mean ± SD	126.8 ± 19.31
Diastolic, mean ± SD	76.39 ± 13.47
**Serum creatinine, mg/dL**	
Valid n	492
mean ± SD	1.46 ± 5.43
**Creatinine clearance, mL/min**	
Valid n	258
mean ± SD	70.5 ± 35.15
**CHA**_**2**_**DS**_**2**_**-VASc, Risk score**	
Valid n	360
mean ± SD	3.3 ± 1.6
**HAS-BLED, Risk score**	
Valid n	269
mean ± SD	1.8 ± 1.2
**Frailty, N (%)**	
Valid n	435
Yes	74 (17%)
No	361 (83%)
**LV ejection fraction, N (%)**	
Valid n	229
≥ 40%	156 (68%)
< 40%	73 (32%)
**Previous history of CV disease, N (%)**	
Valid n	590
Hypertension	457 (78%)
Heart Failure	164 (28%)
Myocardial infarction	66 (11%)
Coronary Heart Disease	78 (13%)
Stroke	52 (9%)
Peripheral Artery Disease	18 (3%)
Transient ischemic attack	16 (2.7%)
**Diabetes mellitus, N (%)**	
Valid n	573
Yes	172 (30%)
No	401 (70%)
**Obesity, N (%)**	
Valid n	588
Yes	147 (25%)
No	441 (75%)
**Dyslipidemia, N (%)**	
Valid n	581
Yes	244 (42%)
No	337 (58%)
**Chronic obstructive pulmonary disease, N (%)**	
Valid n	583
Yes	28 (4.8%)
No	555 (95.2%)
**Previous history of major bleeding, N (%)**	
Valid n	579
Yes	11 (1.9%)
No	568 (98.1%)

*^1^ Unknown and missing data were not considered for the percentage calculation. ^2^ For Previous history of cardiovascular disease, one patient could have more than one previous cardiovascular disease; AF: atrial fibrillation; BMI: body mass index.*

The mean body weight was 77.5 Kg and common comorbidities included hypertension (78%), dyslipidemia (42%), and diabetes (30%).

For almost three-quarters of patients, AF was asymptomatic. The most common symptoms reported among the symptomatic patients were palpitations (52%), dyspnea (43%), and fatigue (32%). Permanent AF was the most frequent type (41%), followed by paroxysmal AF (40%). The median CHA2DS2-VASc score was 3, and the median HAS-BLED score was 2. Overall, 17% of patients perceived to be frail.

### Edoxaban prescription information

More than half (51%) of patients received edoxaban as their first-time treatment for NVAF, and 23% received the medication considering the possibility of improved safety or efficacy. A reduced dose (30mg daily) was given to 30% of patients (Table 2).

**Table 2:** – Edoxaban prescription information

Variables	N = 590
**Edoxaban dosage, N(%)**	
Valid n	590
30mg/once daily	179 (30%)
60mg/once daily	411 (70%)
**Reason for prescription, N(%)**	
Valid n	444
First-time OAC treatment for NVAF	227 (51%)
Expected better efficacy	56 (13%)
Expected better safety	45 (10%)
High variability of former OAC treatment response	35 (8%)
Lack of efficacy of former OAC treatment	13 (3%)
Patient’s request	13 (3%)
Expected better patient compliance	8 (2%)
Drug-drug interaction in the former treatment	2 (0.5%)
Other	45 (10%)
**Interruption of edoxaban, N(%)**	
Number of patients	117 (21%)
Number of interruptions	188 (13%)
Reason for interruption*, N of interruption (%)	
Valid n	188
Adverse event	42 (22%)
Patient’s request	33 (18%)
AF intervention	12 (6%)
Healthcare system related	6 (3%)
Drug-drug interaction	1 (0.5%)
Other	94 (50%)
**Restarting edoxaban after interruption, N (%)**	
Valid n	117
Patients that restarted and ongoing	15 (13%)
Patients that interrupted and did not restart (discontinued)	102 (87%)
**Treatment duration, months**	
Valid n	590
Mean ± SD	10.4 ± 3.8
Median	12.25
IQR (25-75)	5.58 (7.46 - 13.04)

*Reason for discontinuation reported by a unique patient; patient could have more than one interruption; NVAF: non-valvular atrial fibrillation; AF: atrial fibrillation; OAC: oral anticoagulants; SD: standard deviation; IQR: interquartile range.

Treatment was interrupted in 21% of patients, with 188 interruptions recorded throughout the study period. Reasons included adverse events (22%), patient request (18%), AF intervention (6%), healthcare service issues (3%), drug interactions (0.5%), and other reasons (50%). Among these, the most reported were financial issues (10%, N = 18).

After discontinuation, 13% restarted edoxaban, while 87% permanently stopped (Table 2). At the one-year follow-up, 83% of patients were still on edoxaban.

### Safety outcomes

Figure 2 presents the occurrence of major bleeding and CRNMB, according to the International Society on Thrombosis and Haemostasis (ISTH), observed during the one-year of follow-up. A total of 13 primary safety events were registered during the study period; nine major bleeding events were reported, of those five were gastrointestinal bleeds (IP 0.85 [95% CI =0.82; 0.88]), 1 was intramuscular with compartment syndrome (IMCS) (IP 0.17 [95% CI =0.16; 0.18]) and 3 events were classified as others (IP 0.51 [95% CI= 0.48; 0.53]). Regarding the causes of the major bleeding events, six of them were considered spontaneous (IP 1.02 [95% CI= 0.98; 1.05]) and three events had the cause reported as unknown. Among all major bleeding events, 2 (IP 0.34 [95% CI =0.32; 0.36]) of them lead to a fatal outcome.

**Figure 2:** – Bleeding event according to the International Society on Thrombosis and Hemostasis (ISTH) (CRNMB + MB); IP: incidence proportion; CI: confidence interval. Number of bleeding events (N) [IP (95%CI)]

Among the four cases of CRNMB, two were gastrointestinal bleeding (IP 0.34 [95% CI =0.32; 0.36]) and two were classified as others (IP 0.34 [95% CI =0.32; 0.36]). All cases (IP 0.68 [95% CI =0.65; 0.71]) of CRNMB were reported as spontaneous.

Considering all major and CRNM bleeding events (n=5), four were related to the genitourinary system and one was recorded as nasal bleeding (data not shown). No intracranial bleeding was reported during the study period.

Among major and CRNM bleeding cases, nine were related to edoxaban (Table 3). Of these, seven (IP 1.19 [95% CI 1.15;1.22]) were major bleeding events and two (IP 0.34 [95% CI 0.32;0.36]) were CRNMB. Considering the type of bleeding, most of them were gastrointestinal bleeding (N=5; IP 0.85 [95% CI 0.82;0.88]).

**Table 3:** – Bleeding events related to Edoxaban

	N events	N patients	IP (95%CI)
**Bleeding (ISTH)**			
Major bleeding	7	7	1.19 (1.15;1.22)
CRNMB	2	2	0.34 (0.32;0.36)
**Type of bleeding**			
Gastrointestinal	5	5	0.85 (0.82;0.88)
IMCS	1	1	0.17 (0.16;0.18)
Others	3	3	0.51 (0.48;0.53)

*ISTH: International Society on Thrombosis and Hemostasis (ISTH); IP: incidence proportion; CI: confidence interval; CRNMB: Clinically relevant non-major bleeding.*

Twelve minor bleeding events were reported: two gastrointestinal (IP 0.34), one intra-ocular (IP 0.17), and nine others (Table S1). Among other types of minor bleeding events, the most reported one was epistaxis (N = 3) (data not shown). Eleven events (IP 1.86) were caused by spontaneous reasons.

### Effectiveness outcomes

Regarding effectiveness outcomes, Figure 3 presents the incidence of cardiovascular events among AF patients. Effectiveness assessment showed that overall, 112 (23.9%) patients had 141 cardiovascular events (IP 23.9 [95% CI 23.76;24.04]). Four events of stroke (IP 0.68 [CI 95% 0.65;0.71]) were recorded during the study period –three (IP 0.51 [CI 95% 0.48;0.53]) ischemic stroke and one was considered a stroke of unknown cause (IP 0.17 [CI 95% 0.16;0.18]) (data not shown). ACS (N=8; IP 1.36 [CI 95% 1.32;1.39]), TIA (N=1; IP 0.17 [CI 95% (0.16;0.18]) and VTE (N=1; IP 0.17 [CI 95% (0.16;0.18]) had a lower IP compared to other cardiovascular events, representing less than 2% of the patients. No patient presented SEE.

**Figure 3:** – Cardiovascular events reported throughout the study. TIA: transient ischemic attack; VTE: venous thromboembolism; ACS: acute coronary syndrome; MACE: major adverse cardiovascular events; CHF: chronic heart failure.

Congestive heart failure (N=29; IP 4.92 [CI 95% 4.84;4.99]) and MACEs (N=25; IP 4.24 [95% CI 4.17;4.3]) had the highest IP, with approximately 5% of the patients presenting at least one of the events.

Regarding the cardiovascular events related to edoxaban, one TIA (IP 0.17 95% CI [0.16;0.18]) was registered throughout the study.

Overall, 107 patients had 130 events classified as serious adverse events (SAE) (IP 22.03 [95% CI 21.9;22.17]). Among those SAEs, 58 events resulted in death (IP 9.83 [95% CI 9.73;9.93]), however, most of the events (N= 75) resulted in recovery (IP 12.71 [95% CI 12.6;12.82]).

Considering the death occurred during the study, two of them were related to major bleeding events and one was related to TIA (data not shown). Despite the limited number of events observed among study participants, which makes precise time-to-event estimation challenging, Figure 4 presents the Kaplan-Meier analyses of the time (in months) from edoxaban initiation to the occurrence of any cardiovascular events (A). Additionally, it illustrates the Kaplan-Meier estimation from baseline to death related to cardio-vascular events (B).

**Figure 4:** – Kaplan-Meier curves of the time in months from baseline to cardiovascular event (A) and the time from baseline to death (B).

## Discussion

This is a prospective, non-interventional study that provides outcome data of patients treated routinely with edoxaban in Brazilian care centers, enrolled in the EdoBRA study. The findings reinforce the safety and effectiveness of edoxaban, especially in terms of bleeding, and cardiovascular and thromboembolic events in clinical practice.

The study enrolled 705 participants from 30 sites in Brazil. Among them, 590 participants successfully attended at least one follow-up visit or had an event reported, making them eligible for inclusion in the full analysis set. In spite of the fact that EdoBRA was conducted between 2019 and 2023, a period that encompassed the global COVID-19 pandemic, the follow-up mean is in line with the mean period of a similar study conducted in another period of time.^10^

Demographic characteristics of EdoBRA study population are in line with findings from previous studies.^10,12,13^ Additionally, EdoBRA results illustrate that participants were mostly elderly at edoxaban initiation, with a mean age of 68.9 years, which is slightly lower compared to similar populations as in ETNA-AF (mean [±SD] 73.6 ± 9.46)^10^ and ENGAGE AF-TIMI-48 (mean [±SD] 71.4 ± 9.7).^9^ Besides, the current study demonstrated that 18% of participants aged 80 years or older, which is slightly higher than the data from the last National Health Survey conducted in 2013 by the Brazilian Ministry of Health that showed that octogenarians represented 13.6% of the older population (≥ 60 years). This reflects a global trend of an aging population. Brazil, particularly, has one of the largest ageing populations worldwide. According to estimates from the Brazilian Institute of Geography and Statistics 2022 census, there were 4.6 million Brazilians aged 80 and above, representing approximately 2.27% of the total population.^14^

On the other hand, EdoBRA participants in general presented higher proportions of comorbidities, especially heart failure and stroke compared to ETNA-AF study.^10^ Hypertension, a known risk factor for stroke and usually common among AF patients, was the main comorbidity observed in our study population, which is in line with the findings in the literature.^3,9,10^ The aging process and the presence of various comorbidities are associated with an increased risk of thromboembolic and bleeding events in patients with AF.^15^

Considering AF disease characteristics, 40% of EdoBRA patients presented the advanced type of AF (permanent) at baseline. Although these results seem to be higher compared to 22% of the patients reported by ETNA study,^10^ they are in line with the results reported in the ENGAGE AF-TIMI-48 that demonstrated that older patients and those with permanent AF, lower left ventricular ejection fraction, among other characteristics were more likely to die of sudden death or heart failure.^9^ Furthermore, lifestyle-related factors, such as a high body mass index and a history of diabetes, are associated with AF progression. These factors may help explain the elevated percentage of permanent AF observed at baseline in EdoBRA patients.^16^

Almost three-quarters (73%) of EdoBRA patients presented Asymptomatic AF at baseline. The prevalence of asymptomatic presentation among patients with AF has not been established; some studies suggest percentages ranging from 10% to 40% depending on population characteristics.^17^ Regarding AF symptoms, the most frequently reported ones – palpitation, dyspnea and fatigue – are reported to be the most common symptoms related to AF.^18^ Furthermore, the CHA2DS2-VASc and HAS-BLED risk scores, which assess the risk of stroke and bleeding, respectively, demonstrated median scores of 3.3 and 1.8 at baseline. These scores remained consistent throughout the entire study period (data not shown). CHA2DS2-VASc scores value were similar to the findings from ETNA-AF; however, were lower compared to the non-Asian cohort of ENGAGE AF-TIMI-48 (mean [±SD] 4.3 ± 1.41).^9^ Considering the HAS-BLED risk score the values are in line with the ENGAGE AF-TIMI-48 but are lower compared to the ETNA-AF-Europe study population (mean [±SD] 2.5 ± 1.10).^10^ Together, these results indicate a high risk profile for thromboembolic events and a moderate risk for bleeding in the study population.^19,20^

The characteristics of edoxaban prescription indicated that around 30% of patients were prescribed a dosage of 30 mg once daily. About half of the patients were receiving an oral anticoagulant treatment for NVAF for the first time. Regarding edoxaban dosage the criteria for dose reduction (renal impairment, lower body weight, and concomitant P-glycoprotein inhibitor therapy) are markers for patients at high risk of thromboembolic and bleeding events.^21^ Although the numbers support somehow that EdoBRA patients had higher proportions of risk characteristics, the dose prescribed was in line with the literature as 30% of EdoBRA patients were taking 30mg.^9,10^ NOACs dosing remains a challenge in real clinical practice.^21^

Treatment persistence remained high throughout the study period with a total discontinuation rate of 21%. These results are higher than the recent results from ETNA-AF^10^ that demonstrated a discontinuation rate of less than 10%. However, they seem to be in line with other real-world reports that demonstrated discontinuation rates higher than 15% during the first year of follow-up.^22,23^

Among the reasons for edoxaban discontinuation, 18% were related to patients request and 10% related to financial issues. Most of the patients included in the present study depended on public health services, characterizing a low-income population.^24^ Although evidence shows that the rates of NOAC discontinuation are lower compared to VKAs, treatment cost is still a relevant issue regarding NOAC discontinuation.^21^

The EdoBRA study showed low rates of stroke and bleeding events. These results reinforce safety and effectiveness data from a randomized clinical trial on NOACs.^25^ EdoBRA results corroborate the findings from the first international prospective study on patients treated with rivaroxaban (XANTUS registry),^23^ and from the ETNA-AF-Europe, a post-authorization study.^10^ Similar results were also reported in retrospective studies.^26,27^ Results from a large two-year follow-up study also corroborate these findings, showing no significant differences between patients taking both edoxaban dosages.^28^ Similar results concerning the reduction of bleeding events by edoxaban were found in East Asian participants compared to warfarin. The one-year follow-up report of EORP-AF^29^ General Long-Term Registry also demonstrated low rates of stroke and bleeding events in patients treated with NOACs.^29^

Considering all the events occurred during the study, the great majority resulted in patient’s recovery and 58 ended in patient’s death, representing 9.8% of the study population. All-cause mortality rates in patients using edoxaban were low in ETNA-AF report, with a rate of 1.6% yearly.^10^ Latin America cohort in ENGAGE AF-TIMI-48 in use of edoxaban presented an annual mortality rate of more than 6%.^9^ Despite the higher rate of mortality in the EdoBRA study, only three cases were related to bleeding or cardiovascular events (data not shown), which corroborates the results in the literature on patients using edoxaban.^9,10,30^

Edoxaban was proved to reduce AF patients’ mortality mainly due to reduction in bleeding events.^30^ The COVID-19 pandemic is an important factor while analyzing these results not only for the great amount of cumulative death during its period,^31^ but also for its impact on AF follow-up. For example, prescription delays were possibly caused by social mobility restrictions policies. During the pandemic period patients also reported difficulties in scheduling outpatient visits, with an increase in virtual medical appointments.^32^

The strengths of the present study include its prospective design and the lack of restriction to study participation as a characteristic of observational studies, corroborating the generalizability of the research data. Besides, although the study was conducted during COVID-19 pandemic, median follow-up duration is consistent with that in studies conducted in non-pandemic periods.

Although the follow-up rates of the present study are in line with the data shown in the literature, in real-world studies the likelihood of obtaining missing information is lower, since in general, differently from clinical trials, patients’ visits are not scheduled at the research site. Additionally, the study was conducted during the COVID-19 pandemia, which posed significant challenges for patients in scheduling outpatients visits and exams.

Another limitation is the inevitable comparison between the results found in this observational study with clinical trials’ results. Besides, as in the current study most patients were from the northeast region of Brazil, caution is needed in extrapolating the results to the whole population in the country, due to socioeconomic and cultural differences between the regions.

## Conclusion

The EdoBRA study reported low rates for cardiovascular and bleedings events during the 1-year follow-up period, despite the elderly population and accumulation of comorbidities. The results reinforce the effectiveness and the safety of edoxaban in routine treatment of NVAF patients in Brazil.
